# ARE QUALITY INDICATORS IMPORTANT IN COLONOSCOPIES? ANALYSIS OF 3,076 EXAMS IN A PRIVATE TERTIARY SERVICE IN SOUTHEASTERN BRAZIL

**DOI:** 10.1590/0102-6720202400070e1864

**Published:** 2025-02-10

**Authors:** Adriana Borgonovi CHRISTIANO, Danielle Rossana Queiroz Martins BONILHA, Mauro Augusto MARCHIORI, Priscilla de Sene Portel OLIVEIRA, Maria de Lourdes Setsuko AYRIZONO

**Affiliations:** 1 Universidade Estadual de Campinas, Faculty of Medical Sciences, Department of Surgery - Campinas (SP), Brazil; 2 Fundação Centro Médico de Campinas - Campinas (SP), Brazil

**Keywords:** Adenoma, Polyps, Colorectal Neoplasms, Carcinogenesis, Colonoscopy., Adenoma, Pólipos, Neoplasias Colorretais, Carcinogênese, Colonoscopia.

## Abstract

**BACKGROUND:**

The carcinogenesis of colorectal cancer is well understood. Adenomas are the precursor lesions in about 70% of cases, highlighting the importance of screening programs.

**AIMS:**

The aim of this study was to analyze the effectiveness of colonoscopy examinations performed in a private tertiary service by calculating the polyp detection rate (PDR) and adenoma detection rate (ADR) and comparing these rates with literature data.

**METHODS:**

This retrospective observational study evaluated colonoscopies performed at Hospital Centro Médico de Campinas between 2018 and 2020. It assessed the indications and complications of colonoscopy, sex, age group, bowel preparation, cecal intubation rate, ADR, PDR, and advanced adenoma detection rate (AADR).

**RESULTS:**

During the period, 3,686 colonoscopies were performed, and 3,076 were included in the analysis. The mean patient age was 57.2 years, and most patients were female (53.5%). Complications occurred in 39 colonoscopies (1.3%), with bleeding in six cases and perforation in one case. Tubular adenoma was the most prevalent histological subtype found in 20% of tests and in 62.7% of those with positive findings. The PDR was 23% and significantly increased with advancing age (p<0.01). The ADR was 20% and also significantly increased with age (p<0.001). This rate was higher in men (27%). The AADR was 4%.

**CONCLUSIONS:**

Colonoscopy is an effective polyp detection method, and the PDR was higher in men and significantly increased with age. The ADR and AADR were comparable to the literature data.

## RESUMO

**RACIONAL:**

A carcinogênese do câncer colorretal é conhecida, sendo os adenomas as lesões precursoras em cerca de 70% dos casos, destacando-se a importância dos programas de rastreamento.

**OBJETIVOS:**

Analisar a eficácia dos exames de colonoscopia realizados em um serviço terciário, privado, por meio do cálculo da taxa de detecção de pólipos (*polyp detection rate -* PDR) e de adenomas (*adenoma detection rate -* ADR).

**MÉTODOS:**

Estudo retrospectivo observacional de colonoscopias realizadas no Hospital Centro Médico de Campinas, entre 2018 e 2020. Foram analisados: indicação do exame, sexo, faixa etária, preparo de cólon, complicações, taxa de intubação cecal, PDR, ADR e taxa de detecção de adenomas avançados (*advanced adenoma detection rate -* AADR).

**RESULTADOS:**

No período, foram realizadas 3.686 colonoscopias, com 3.076 exames incluídos para análise. Média de idade foi de 57,2 anos, sendo 53,5% do sexo feminino. Complicações ocorreram em 39 colonoscopias (1,3%), sendo 6 casos de sangramento e uma perfuração intestinal. Adenoma tubular foi o subtipo histológico mais prevalente, encontrado em 20% dos exames e em 62,7% daqueles com achados de alguma lesão. A PDR foi de 23%, com aumento significativo com o aumento da faixa etária (p<0,01) e ADR de 20%, também significativamente maior na idade avançada (p<0,001), sendo superior em homens (27%), enquanto a AADR foi de 4%.

**CONCLUSÕES:**

A colonoscopia constituiu um método eficaz na detecção de pólipos, sendo a PDR maior em homens, aumentando significativamente com a progressão da faixa etária. A ADR e a AADR foi comparável com as da literatura.

## INTRODUCTION

Colorectal cancer (CRC) is the third most common neoplasm among men and women worldwide^44^. In Brazil, CRC ranks third in cancer-related mortality and second in incidence among males and females^10^. Since its process of carcinogenesis is known, screening for this neoplasm is feasible^4^. Adenomas account for 70% of sporadic CRC cases, while serrated lesions account for 25-30%^9,45^.

The success of screening programs is demonstrated by the reduction in the incidence of the disease and associated morbidity/mortality as a result of the early identification and treatment of lesions^4,33,39^. However, Brazil does not have a well-established screening program^26^. The official recommendation is to begin CRC screening in average-risk individuals at age 50^10^, with colonoscopy being the preferred screening test. In the long term, this method is expected to reduce the incidence of CRC by 31-71% and mortality by 65-88% through the identification and treatment of precursor lesions^39^.

Specific quality criteria should be adopted to ensure the effectiveness of colonoscopies, including good colon preparation in more than 90% of tests, a cecal intubation rate=95%, a withdrawal time >6 min, a significant adenoma detection rate (ADR) and sessile serrated polyp detection rate (SSPDR), an adequate resection technique, use of high-resolution imaging, and appropriate surveillance protocols for identified lesions^23,25^.

The ADR is defined as the percentage of colonoscopies in which at least one adenoma is identified and has been accepted as the primary quality indicator for these tests^19,20^. Other metrics such as the polyp detection rate (PDR), advanced adenoma detection rate (AADR), and SSPDR may also be used^31^. This study aimed to evaluate the quality of colonoscopies performed in a private tertiary service in the interior of São Paulo State by calculating ADR, AADR, and PDR and by comparing the results with literature data.

## METHODS

This retrospective observational study involved individuals referred for colonoscopy for CRC screening, polyp follow-up, inflammatory bowel disease monitoring, and symptom investigation (abdominal pain, change in bowel habits, rectal bleeding, and anemia). The examinations were conducted at the Colonoscopy Service of Hospital Centro Médico de Campinas, Campinas (SP), from January 2018 to January 2020.

Patients between 18 and 85 years were included in the study. Exclusion criteria were missing colonoscopy and histopathological data, inadequate bowel preparation (Boston Scale <6), examinations lasting less than 10 min or performed on an emergency basis, active endoscopic inflammatory bowel disease, cases referred for therapeutic procedures (resection of pre-identified lesions, endoscopic dilation, treatment of surgical complications), prior total colectomy, and incomplete examination, except for cases of stenosing neoplasia.

Bowel preparation consisted of administering 500 mL of a 10% mannitol solution or three sachets of sodium picosulfate (Picoprep^®^) combined with a clear liquid diet on the day before the test. Colon preparations were assessed using the Boston Bowel Preparation Scale in examinations conducted after January 2019, when this scale was adopted by the service. All procedures were performed using Olympus CF-Q180AL and CF-H170L video colonoscopies. The following clinical and demographic characteristics of the participants were analyzed: age, sex, colonoscopy indication, total examination time, and complications.

Lesions in the cecum, ascending, and transverse colon were classified as proximal; lesions in the descending colon, sigmoid, and rectum were classified as distal. Based on the histopathological findings, polyps were classified as hyperplastic, serrated adenoma, tubular adenoma, villous or tubulovillous adenoma, and adenocarcinoma. The Vienna classification was used to define the degree of dysplasia^35^. Lesions ≥10 mm, in the presence of a villous component or high-grade dysplasia, were defined as advanced adenomas^11^. Pathologists from two laboratories in Campinas (SP) provided the pathology reports according to the examiners’ preferences.

To describe the profile of the sample, frequency tables of the categorical variables were created, calculating the mean and standard deviation (SD), and absolute and relative frequency. A test of proportions was used to compare lesion detection rates between the screening group and the group of other indications. A test for trend in proportions was applied to compare lesion detection rates among different age groups. A level of significance of 5% was adopted. The analyses were performed using R 2023 (R: A Language and Environment for Statistical Computing. R Foundation for Statistical Computing, Vienna, Austria; https://www.R-project.org/).

The study was approved by the Research Ethics Committee of the Faculty of Medical Sciences, Universidade Estadual de Campinas (number: 5.084.635 and CAAE: 52244821.9.0000.5404), and was conducted in accordance with the Declaration of Helsinki.

## RESULTS

Data from 3,686 colonoscopies were collected, and 610 exams were excluded. Inadequate bowel preparation (n=149), incomplete data (n=113), and examinations performed on an emergency basis (n=70) were the main reasons for exclusion. The final sample consisted of 3,076 colonoscopies. There were 53.5% of females, and the mean age was 57.2 years (SD=13.1) (Table 1). The cecal intubation rate was 97.4%, and the mean total examination time was 13.6 min. Cecal intubation and withdrawal times were recorded for 161 colonoscopies, with mean times of 8.47 and 6.14 min, respectively. The Boston Bowel Preparation Scale was assessed in 952 colonoscopies, and the mean score was 8.9 (Table 1).

**Table 1:** Sociodemographic and colonoscopy characteristics.

Variable	Mean (SD) or N (%)	N total
Age (years)	57.2 (13.1)	3,076
Total examination time (min)	13.6 (6.63)	3,076
Cecal intubation time (min)	8.47 (4.29)	161
Withdrawal time (min)	6.14 (4.25)	161
Boston Scale	8.90 (0.58)	952
Sex		
Female	1,646 (53.5)	3,076
Male	1,430 (46.5)	
Age group (years)		
<30	95 (3.1)	3,076
30-45	391 (12.7)	
45-50	280 (9.1)	
=50	2,310 (75.1)	
Complication		
No	3,037 (98.7)	3,076
Yes	39 (1.3)	
Type of complication		
Abdominal pain	24 (55.8)	43^^*^^
Fever	7 (16.3)	
Bleeding	6 (14.0)	
Nausea and vomiting	4 (9.3)	
Bowel perforation	1 (2.3)	
Other	1 (2.3)	

SD: standard deviation.

* One individual can have more than one type of complication.

Complications were reported in 39 colonoscopies (1.3%) and abdominal pain requiring analgesia was the most frequent (55.8%). Bleeding occurred in six examinations (13.9%). There was one case of intestinal perforation (2.3%) (Table 1). Complications were defined as those occurring within 30 days of the procedure. All cases of bleeding ceased spontaneously; however, one patient required a revisional colonoscopy with endoclip placement at the polypectomy site. The case of intestinal perforation was treated by laparoscopic rectosigmoidectomy with a satisfactory outcome.

A total of 756 adenomas were identified. Tubular adenoma was the most prevalent subtype, observed in 20% of all colonoscopies and in 62.7% of those with positive findings. Additionally, 191 hyperplastic polyps and 61 serrated adenomas (serrated sessile lesions by the current classification) were identified, corresponding to one-quarter of the lesions in positive tests. Additionally, 13 in situ adenocarcinomas and 4 advanced adenocarcinomas were also detected (Table 2).

**Table 2:** Histopathological study.

Subtype	N	% of total	% of positive tests
Tubular adenoma	622	20.2	62.7
Tubulovillous adenoma	132	4.3	13.3
Villous adenoma	2	0.1	0.2
Serrated adenoma	61	2.0	6.1
Hyperplastic	191	6,2	19.3
In situ adenocarcinoma	13	0.4	1.3
Advanced adenocarcinoma	4	0.1	0.4
Nonspecific colitis	24	0.8	2.4
Gastrointestinal stromal tumor	2	0.1	0.2
Lipoma	5	0.2	0.5
Other	13	0.4	1.3

In total, 203 flat lesions were identified, with a mean size of 13.7 mm (SD=7.62 mm). There were 567 sessile polyps, with a mean size of 5.5 mm (SD=3.33 mm). The mean size of pedunculated polyps was 15.6 mm (SD=7 mm), while semi-pedunculated polyps had a mean size of 11 mm (SD=3.8 mm). Tubular adenoma was the most frequent histological subtype among all morphological types. The highest prevalence of lesions was observed in the sigmoid colon, accounting for 36% of positive tests.

The overall PDR was 23% (28% in men and 20% in women). This rate was 5% in individuals younger than 30 years but 26% in those aged 50 years and older. Polyps were detected in 30% of examinations of men aged 50 years. A statistically significant association (p<0.001) was observed between PDR and age groups (Table 3). The PDR was 27% in the screening group and 10% in the group of other indications, with the difference being statistically significant (p<0.001) (Table 3).

**Table 3:** Polyp detection rate.

Stratification	N	Total	%	Overall		Among positive tests	
				NPC	SD	MNPC	SD
Total	718	3,076	23	0.38	0.91	1.62	1.23
Female (F)	323	1,646	20	0.30	0.78	1.53	1.11
Male (M)	395	1,430	28	0.47	1.03	1.70	1.31
<30 years	5	95	5	0.05	0.22	1.00	-
30-45 years	58	391	15	0.20	0.60	1.34	0.93
45-50 years	49	280	18	0.23	0.55	1.31	0.55
=50 years	606	2,310	26	0.44	0.99	1.68	1.29
F: <30	4	61	7	0.07	0.25	1.00	-
F: 30-45	29	230	13	0.17	0.62	1.38	1.18
F: 45-50	18	150	12	0.17	0.52	1.44	0.62
F: =50	272	1,205	23	0.35	0.84	1.56	1.14
M: <30	1	34	3	0.03	0.17	1.00	-
M: 30-45	29	161	18	0.24	0.57	1.31	0.60
M: 45-50	31	130	24	0.29	0.58	1.23	0.50
M: =50	334	1,105	30	0.54	1.12	1.78	1.39
Distal	358	403	89	1.14	0.78	1.28	0.71
Proximal	193	261	74	0.90	0.69	1.21	0.51
Proximal and distal	160	164	98	2.81	1.84	2.88	1.80
Screening	652	2,414	27	0.44	0.98	1.64	1.26
Other indication	66	662	10	0.14	0.51	1.41	0.89

NPC: number of polyps per colonoscopy; SD: standard deviation p<0.001; MNPC: mean number of polyps per colonoscopy.

The overall ADR was 20%. When stratified by age, the ADR was 1% in individuals younger than 30 years, 11% in those aged 30-45 years, 15% in those aged 45-50 years, and 23% in individuals over 50 years (Table 4). A statistically significant association was observed between ADR and age group, with a higher older age group (p<0.001) (Table 4). When stratified by sex, the ADR was 17% in women and 24% in men. Considering sex and age, the ADR was 20% in women and 27% in men over 50 years (Table 4). Considering only CRC screening, the ADR was 23% versus 9% for other indications. This difference was also statistically significant (p<0.001) (Table 4).

**Table 4:** Adenoma detection rate.

Stratification	N	Total	%	Overall		Among positive tests	
				Average	SD	MNAC	SD
Total	622	3,076	20	0.25	0.55	1.22	0.52
Female (F)	276	1,646	17	0.20	0.47	1.17	0.44
Male (M)	346	1,430	24	0.31	0.61	1.27	0.58
<30 years	1	95	1	0.01	0.10	1.00	-
30-45 years	42	391	11	0.12	0.38	1.14	0.42
45-50 years	42	280	15	0.17	0.42	1.12	0.33
=50 years	537	2,310	23	0.29	0.59	1.24	0.54
F: <30	1	61	2	0.02	0.13	1.00	-
F: 30-45	23	230	10	0.11	0.34	1.09	0.29
F: 45-50	10	150	7	0.08	0.32	1.20	0.42
F: =50	242	1,205	20	0.24	0.51	1.18	0.45
M: <30	0	34	0	-	-	-	-
M: 30-45	19	161	12	0.14	0.43	1.21	0.54
M: 45-50	32	130	25	0.27	0.50	1.09	0.30
M: =50	295	1,105	27	0.34	0.65	1.29	0.60
Distal	132	403	33	0.73	0.57	1.08	0.31
Proximal	68	261	26	0.84	0.60	1.13	0.38
Proximal and distal	10	164	6	1.49	0.83	1.59	0.76
Screening	561	2,414	23	0.29	0.58	1.22	0.52
Other indication	61	662	9	0.11	0.39	1.21	0.52

SD: standard deviation p<0.001; MNAC: mean number of adenomas per colonoscopy.

Adenomas were more frequently detected in the distal segments, descending colon, sigmoid, and rectum, accounting for 33% of all lesions. The mean number of adenomas per colonoscopy, calculated from colonoscopies with one or more adenomas, was 1.22. Advanced adenomas were detected in 3% of the tests and were more frequent in men over 50 years. In this study, no advanced adenomas were found in individuals under 30 years of age. Considering only tests performed for screening purposes, the AADR was 4% (Table 5). There was also a predominance of these lesions in distal segments.

**Table 5:** Advanced adenoma detection rate.

Stratification	N	Total	%	Overall		Among positive tests	
				Mean	SD	Mean	SD
Total	95	3,076	3	0.03	0.20	1.09	0.33
Female (F)	37	1,646	2	0.02	0.17	1.08	0.28
Male (M)	58	1,430	4	0.04	0.23	1.10	0.36
<30 years	0	95	0	-	-	-	-
30-45 years	8	391	2	0.02	0.17	1.12	0.35
45-50 years	6	280	2	0.02	0.15	1.00	-
=50 years	81	2,310	4	0.04	0.21	1.10	0.34
F: <30	0	61	0	-	-	-	-
F: 30-45	4	230	2	0.02	0.13	1.00	-
F: 45-50	3	150	2	0.02	0.14	1.00	-
F: =50	30	1,205	2	0.03	0.18	1.10	0.31
M: <30	0	34	0	-	-	-	-
M: 30-45	4	161	2	0.03	0.21	1.25	0.50
M: 45-50	3	130	2	0.02	0.15	1.00	-
M: =50	51	1,105	5	0.05	0.24	1.10	0.36
Distal	38	403	9	0.10	0.33	1.08	0.27
Proximal	22	261	8	0.09	0.30	1.05	0.21
Proximal and distal	35	164	21	0.24	0.51	1.14	0.43
Screening	86	2,414	4	0.04	0.21	1.08	0.28
Other indication	9	662	1	0.02	0.16	1.22	0.67

SD: standard deviation.

Hyperplastic polyps were observed in 6% of the tests, with a statistically significant difference between examinations performed for screening purposes (7%) and other indications (2%) (p<0.001). A statistically significant association was also found between hyperplastic polyps and age group, with higher rates observed in older age groups (p<0.001). The detection rate of serrated adenomas was 2%, with no significant difference between sexes. No serrated adenomas were detected in individuals under 30 years of age, and there were no significant differences among the various age groups. Malignant neoplasms were detected in 17 tests, with no significant differences between sexes. Malignancies were more common in individuals over 50 years.

## DISCUSSION

Colonoscopy is an operator-dependent procedure. Factors that influence lesion detection include bowel preparation, withdrawal time, endoscopist experience, devices that increase mucosal exposure, and imaging technologies^1,3,15,39,41^. This study described the pattern of colonoscopies performed in a private tertiary hospital in the interior of the State of São Paulo. The sample consisted of individuals seen at a private service, who were not users of the Unified Health System (Sistema Único de Saúde - SUS), and who were referred by their physicians. The results obtained may reflect the fact that CRC screening programs have not yet been fully established in Brazil. Despite awareness of the need for prevention measures, access to specialists, particularly within SUS, is limited, impairing the correct application of guidelines for the follow-up of detected lesions^6,14^.

In this study, the cecal intubation rate was 97%, consistent with recommended guidelines^23^. In addition, the complication rate (1.3%) was low in agreement with the main meta-analyses reported in the literature^24,30^. However, a limiting factor in the assessment of complications was that only cases of individuals who sought emergency care at the hospital were identified, since these events are reported in the medical. The main complications, such as bleeding and perforation, were associated with therapeutic procedures, in which these rates tend to be higher^24,30^.

The ADR is the percentage of colonoscopies with at least one identifiable adenoma and is accepted as the primary quality indicator for these tests^19,20^. Corley et al.^8^ demonstrated a reduction in interval cancer with increasing ADR. The national literature is scarce, and consensus on the ideal Brazilian ADR, a country with a mixed population, continental size, and cultural variability among its different regions, is still needed. Studies conducted at services in the southern and central-western regions of the country reported ADRs that are consistent with the international literature^7,13,26,29^.

The overall ADR was 20%. Rates ranging from 5 to 37.5% have been reported in the literature^21^, with recommendations of about 25% for mixed samples of men and women^34^. Possible factors that may have contributed to the rate observed here include the predominance of females (53.5%), the number of individuals under 50 years, and the indication and interval of colonoscopies. A predominance of women has also been observed in other national studies^7,13,26,27,29^. Culturally, Brazilian women are more likely to seek prevention programs or be referred for colonoscopy by their gynecologists^7^. Additionally, according to the latest Brazilian Institute of Geography and Statistics (Instituto Brasileiro de Geografia e Estatística - IBGE) census, there is a predominance of women in several regions of the country, whose life expectancy is higher than that of men (79 versus 72 years)^17^. Lower ADRs are expected for women^34^, and the predominance of females in the sample may therefore have contributed to the overall rate found. Another Brazilian study with female predominance reported a lower ADR among women^26^. Male sex is considered an independent risk factor for increased ADR^38^. In the present sample, the ADR was 24% among males, but 17% among females. However, when only screening colonoscopies in individuals=50 years were considered, the ADR was 20% among women and 27% among men, with an overall rate of 23%, values that are within current recommendations^31^.

Age is another independent risk factor for ADR, with higher rates being observed in individuals over 50 years. In our study, the increase in ADR with age was statistically significant, consistent with literature data^38^. Following the change in the United States CRC screening guidelines starting at age 45, studies are being conducted to determine the ADR in the 45-49 age group. There is a trend toward a slightly lower ADR in this group than in the group of 50-54 years^32^. Bilal et al.^5^ observed an ADR of 28% in the 45-49 age group compared to 38% in the 50-54-year-old group. In our study, the ADR was 15% in the 45-49 age group, but 25% among males, a value slightly lower than that found in men over 50 years of age. Moura et al.^26^ also observed an ADR of about 25% in the 45-49 age group. This is an important finding since the recommended starting age of CRC screening in Brazil is still 50 years for the average-risk population. One-quarter of our sample consisted of individuals under 50 years old, a fact that may have contributed to the lower overall ADR found. Shaukat et al.^40^ estimated that, if the percentage of screening colonoscopies in younger patients (<50 years) at a service is 10 and 25%, a decrease in ADR of 1% and 3%, respectively, is expected.

The indication of colonoscopy is also essential in determining the ADR, which tends to be higher in surveillance colonoscopies than in screening tests^32,38^. Identifying the number of index colonoscopies in the sample was not possible, with the overall ADR being 23% in the screening group. Although recent literature suggests that including diagnostic tests in the ADR calculation is insufficient to lower the recommended thresholds, a statistically significant difference in ADR was found between the screening and other indication groups^32^.

Adopting international follow-up guidelines is considered a quality criterion for colonoscopies^14,23,32,33^. The inadequate application of these recommendations can lead to unnecessary expenses and additional patient risk^11^. Subsequent colonoscopies in the same individual were not identified for evaluation of routine surveillance procedures due to the service profile, which performs examinations requested by different general practitioners or specialists. Unlike done in the United States, monitoring the excessive use of colonoscopies for average-risk individuals is not common in Brazil^11^.

The PDR is easy to obtain and correlates with the ADR, as demonstrated in previous studies^12,43^. Another advantage is that its calculation does not require histopathological examination^2^. However, some authors advocate against its use as a quality parameter, arguing that removing nonsignificant polyps like hyperplastic ones in the rectosigmoid can easily skew the results^18,31^. In this case series, the overall PDR was 23%, with a rate of 28% among men and 20% among women. There was a statistically significant increase in PDR with increasing age, consistent with other studies^22,36,42^.

On the contrary, the AADR reported in the literature ranges from 4 to 10%^28^. In a cohort of 200,000 colonoscopies, Penz et al.^28^ demonstrated a correlation between AADR and ADR, with the former increasing proportionally. Furthermore, the AADR does not vary significantly between highand low-performance endoscopists, with a 25% ADR cutoff. The use of AADR as a quality criterion remains controversial, since lesion size tends to vary between observers^8^.

The detection rate of sessile serrated lesions is variable among endoscopists, even among high-performing ones^16,37^. There is still a lack of consensus among pathologists on the classification of serrated lesions, even after the 2010 revision^6^. We therefore did not include SSPDR as a quality criterion in the analysis. In our service, specimens are sent to two different pathology laboratories in the city according to the preference of each endoscopist. Both laboratories have used the previous WHO classification for sessile lesions, explaining the term “serrated adenoma” used in this study. It is possible that some of the hyperplastic polyps were in fact serrated lesions.

Continuous education and training of professionals are essential for improving examination quality and for maintaining low complication rates. Periodic revision of the results is recommended to improve ADR and AADR. Assessment of the SSPDR should also be encouraged, including efforts to standardize the classification of serrated lesions among pathologists and to improve the evaluation of the proximal segments of the colon^25^.

This study has significant limitations, mainly due to its retrospective design; however, it reports the findings of a private colonoscopy service with extensive experience in this procedure. The principal investigator collected all data, which helped reduce potential biases. Prospective studies involving robust case series are needed to obtain more detailed conclusions regarding the ideal ADR, AADR, and SSPDR in Brazil.

## CONCLUSIONS

Colonoscopy proved to be an effective method for detecting polyps and adenomas with a low complication rate. The PDR was higher among men and increased significantly with advancing age. The ADR and AADR were comparable to those reported in the literature. Tubular adenomas predominated in the distal segments of the colon, while adenocarcinomas were not frequent.

## Central Message

Colorectal cancer (CRC) ranks third in cancer-related mortality and second in incidence among males and females in Brazil. The official recommendation is to begin CRC screening in average-risk individuals at age 50, with colonoscopy being the preferred screening test. To ensure the effectiveness of colonoscopies, specific quality criteria, including good colon preparation, a high cecal intubation rate, a withdrawal time >6 min, a significant polyps detection rate (PDR) and adenoma detection rate (ADR), and sessile serrated polyp detection rate (SSPDR), should be established. Additionally, the use of an adequate resection technique, high-resolution imaging, and appropriate surveillance protocols for identified lesions are essential.

## Perspectives

Colonoscopy proved to be an effective method for detecting polyps and adenomas with a low complication rate. The PDR was higher among men and increased significantly with advancing age. The ADR and AADR were comparable to those reported in the literature. Tubular adenomas predominated in the distal segments of the colon, while adenocarcinomas were not frequent.
